# Predictive model identifies key network regulators of cardiomyocyte mechano-signaling

**DOI:** 10.1371/journal.pcbi.1005854

**Published:** 2017-11-13

**Authors:** Philip M. Tan, Kyle S. Buchholz, Jeffrey H. Omens, Andrew D. McCulloch, Jeffrey J. Saucerman

**Affiliations:** 1 Department of Biomedical Engineering, University of Virginia, Charlottesville, Virginia, United States of America; 2 Departments of Bioengineering and Medicine, University of California San Diego, La Jolla, California, United States of America; Johns Hopkins University, UNITED STATES

## Abstract

Mechanical strain is a potent stimulus for growth and remodeling in cells. Although many pathways have been implicated in stretch-induced remodeling, the control structures by which signals from distinct mechano-sensors are integrated to modulate hypertrophy and gene expression in cardiomyocytes remain unclear. Here, we constructed and validated a predictive computational model of the cardiac mechano-signaling network in order to elucidate the mechanisms underlying signal integration. The model identifies calcium, actin, Ras, Raf1, PI3K, and JAK as key regulators of cardiac mechano-signaling and characterizes crosstalk logic imparting differential control of transcription by AT1R, integrins, and calcium channels. We find that while these regulators maintain mostly independent control over distinct groups of transcription factors, synergy between multiple pathways is necessary to activate all the transcription factors necessary for gene transcription and hypertrophy. We also identify a PKG-dependent mechanism by which valsartan/sacubitril, a combination drug recently approved for treating heart failure, inhibits stretch-induced hypertrophy, and predict further efficacious pairs of drug targets in the network through a network-wide combinatorial search.

## Author summary

Common stresses such as high blood pressure or heart attack can lead to heart failure, which afflicts over 25 million people worldwide. These stresses cause cardiomyocytes to grow and remodel, which may initially be beneficial but ultimately worsen heart function. Current heart failure drugs such as beta-blockers counteract biochemical cues prompting cardiomyocyte growth, yet mechanical cues to cardiomyocytes such as stretch are just as important in driving cardiac dysfunction. However, no pharmacological treatments have yet been approved that specifically target mechano-signaling, in part because it is not clear how cardiomyocytes integrate signals from multiple mechano-responsive sensors and pathways into their decision to grow. To address this challenge, we built a systems-level computational model that represents 125 interactions between 94 stretch-responsive signaling molecules. The model correctly predicts 134 of 172 previous independent experimental observations, and identifies the key regulators of stretch-induced cardiomyocyte remodeling. Although cardiomyocytes have many mechano-signaling pathways that function largely independently, we find that cooperation between them is necessary to cause growth and remodeling. We identify mechanisms by which a recently approved heart failure drug pair affects mechano-signaling, and we further predict additional pairs of drug targets that could be used to help reverse heart failure.

## Introduction

Cardiac mechano-signaling, the ability of the heart to sense and respond to mechanical cues, plays an integral role in driving ventricular hypertrophy and remodeling [1,2]. Although hypertrophic remodeling initially functions as a compensatory response to extra workload, the dramatic growth of the ventricles ultimately engenders further cardiac deterioration [3]. Current therapies such as beta blockers and angiotensin II receptor blockers (ARBs) seek to block the chemical ligands initiating hypertrophy in addition to their direct hemodynamic effects [4]. As heart failure worsens, however, many patients become refractory to neurohormonal inhibition, and increased mechanical stretch of the myocytes can stimulate cardiac remodeling independently of the patient’s biochemical status [5,6]. Abnormal ventricular geometry in turn increases the mechanical burden, further heightening wall stress. A better understanding of cardiac mechano-signaling is crucial for identifying therapies that can interrupt this downward spiral [7].

While many mechano-sensitive proteins have been identified in cardiomyocytes [8,9], the mechanisms whereby the downstream signaling cascades are integrated into the hypertrophic response remain unknown [10,11]. Computational models can accelerate insight into complex signaling networks [12], and influential network hubs have previously been identified using logic-based models of biochemically-initiated hypertrophy signaling [13,14]. Past studies of mechano-sensing have used finite element or force dipole models to predict concentric or eccentric cardiac growth [15], to identify the mechanisms coordinating beating between adjacent myocytes [16,17], and to gain insights into force transmission between contracting cells [18]. Others have developed mass-action kinetic models of individual stretch-sensitive pathways to study calcium dynamics [19], or to study TGF-β release in response to substrate stiffness [20]. These approaches, however, have not been used to examine systems-level properties of the signaling network itself.

In this study, we constructed and validated the first computational model of the cardiac mechano-signaling network in order to predict key signaling regulators integrating the stretch-induced hypertrophic response. Synthesizing the current understanding of mechanically driven signaling cascades, the model identifies signaling motifs and crosstalk logic crucial to network function. In particular, coordination between AT1R, integrins, and calcium channels was found to be essential for increased cell size, protein synthesis, and upregulation of the fetal gene program in response to mechanical stress. Rather than converging on a common set of nodes, each mechano-responsive pathway contributes to the cellular response through a distinct group of transcription factors. The model also elucidates cGMP-dependent cooperative mechanisms underlying valsartan/sacubitril, the combination angiotensin receptor–neprilysin inhibitor recently approved for treating heart failure. Combined responses to inhibition or activation of every pair of nodes in the network are then calculated, predicting additional combinations of drug targets with maximal influence over stretch-induced remodeling.

## Results

### A predictive computational model of the cardiomyocyte mechano-signaling network

**Fig 1:**
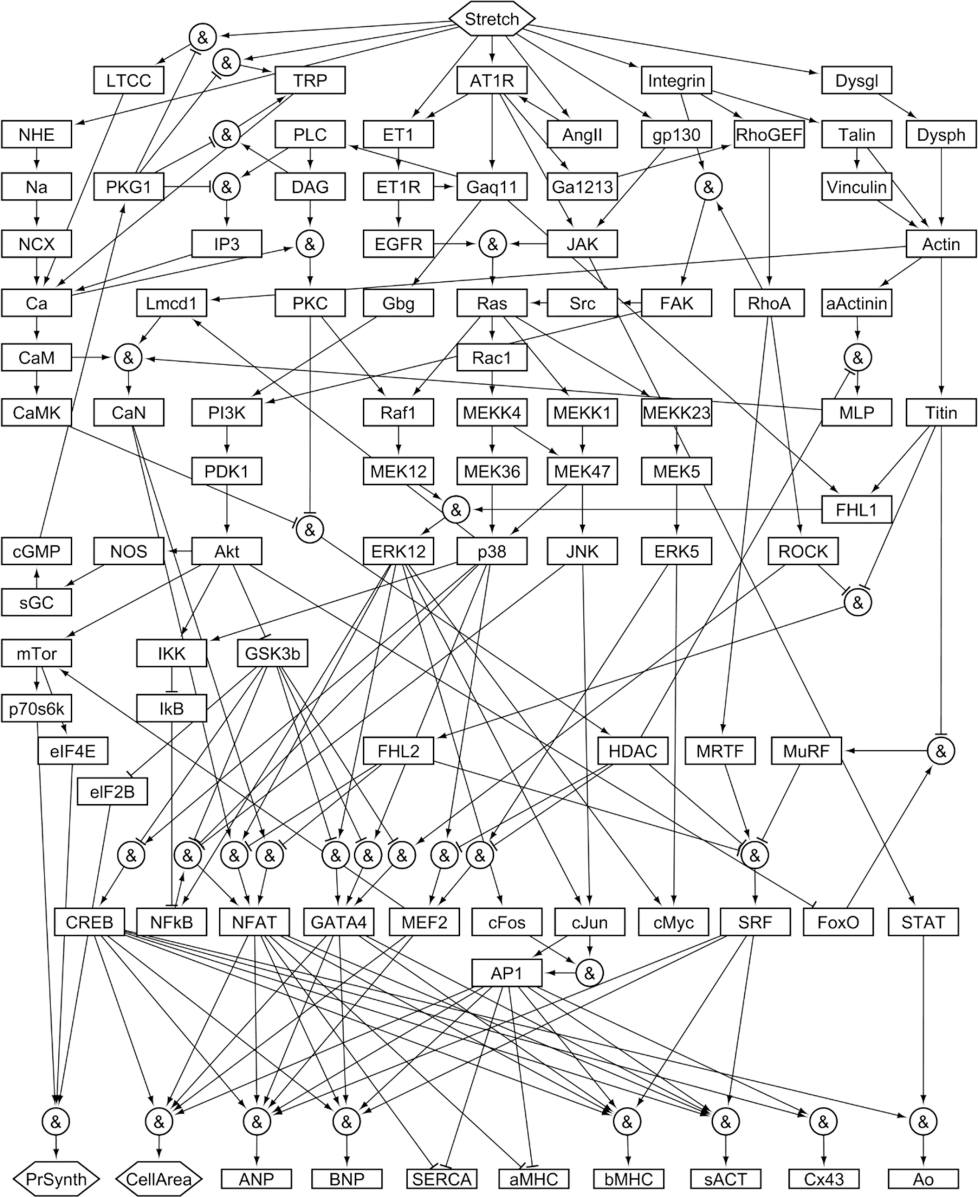
Reconstruction of the mechano-signaling network in cardiac myocytes. The model comprises 125 activating or inhibitory reactions linking 94 nodes, beginning with 9 mechano-sensors (NHE, LTCC, TRP, ET1, AT1R, AngII, gp130, Integrin, and Dysgl) and proceeding through multiple signaling cascades and transcription factors (penultimate row) to 10 hypertrophy-related gene products or phenotypes (final row). Complete lists of model reactions and of abbreviations for node names are provided in S1 Table.

To reconstruct the cardiomyocyte mechano-signaling network (Fig 1), experimental observations were collected from published literature. During literature review, papers involving *in vitro* cell stretching experiments performed in rat cardiomyocytes were set aside for validation, while remaining papers were used to reconstruct the signaling network. In all, a group of 172 papers designated for model construction was used to define network architecture (S1 Table), and a separate group of 55 papers designated for model validation was used to validate model predictions of network activity (S2 Table), an approach used in previous network reconstructions [13,14].

The network incorporates five mechano-sensors each shown to be directly responsive to physical stretch: AT1R (angiotensin type 1 receptor) [8], LTCC (L-type calcium channel) [21], TRP (transient receptor potential channel) [22], integrin [23], and dystroglycan [24]. Also represented are four proteins known to be mechano-responsive, but whose mechanism of stretch-induced activation or release is unknown or disputed: gp130 (glycoprotein 130) [25], NHE (sodium–hydrogen exchanger) [26], Ang II (angiotensin II) [27], and ET-1 (endothelin 1) [28]. Signal propagation continues through downstream mechano-responsive proteins known to be regulated by these mechano-sensors, such as MAPKs (mitogen-activated protein kinases), Akt (protein kinase B), CaN (calcineurin), and FAK (focal adhesion kinase). These proteins in turn activate various transcription factors regulating the 10 phenotypic outputs most commonly reported in the literature, including protein synthesis, cell area, and expression of eight genes: ANP (atrial natriuretic peptide), BNP (brain natriuretic peptide), SERCA (sarcoplasmic reticulum Ca^2+^ ATPase), α-MHC (α-myosin heavy chain), β-MHC (β-myosin heavy chain), sACT (skeletal α-actin), Cx43 (connexin 43), and Ao (angiotensinogen). Activation of the fetal gene program, a hallmark of cardiac stress, encompasses upregulation of ANP, BNP, β-MHC, and sACT, and downregulation of SERCA and α-MHC [29]. In all, the reconstructed network of cardiomyocyte mechano-signaling includes 94 nodes (cytokines, proteins, mRNA, and cell processes), connected by 125 reactions. Further details of network reconstruction are included in the methods.

To convert the network into a predictive computational tool, we modeled reactions with logic-based differential equations (LDEs), a strategy previously used to combine the strengths of mass action kinetic and Boolean models for large-scale networks [30,13,14]. In this approach, the normalized activation of each node (such as phosphorylation for proteins, or expression for mRNAs) is represented by ordinary differential equations with saturating Hill functions, and continuous logical AND or OR logic gates are used to represent pathway crosstalk. In general, OR gating is used when each input to a node is sufficient but not necessary for activation, whereas AND gating is used when each input is necessary. As in previously published models [13,14,30], uniform default values were used for all network parameters. Preservation of network predictions to these constraints has been previously demonstrated [13,14,31], although individual parameters can be tuned when necessary by fitting to experimental measurements [32].

**Fig 2:**
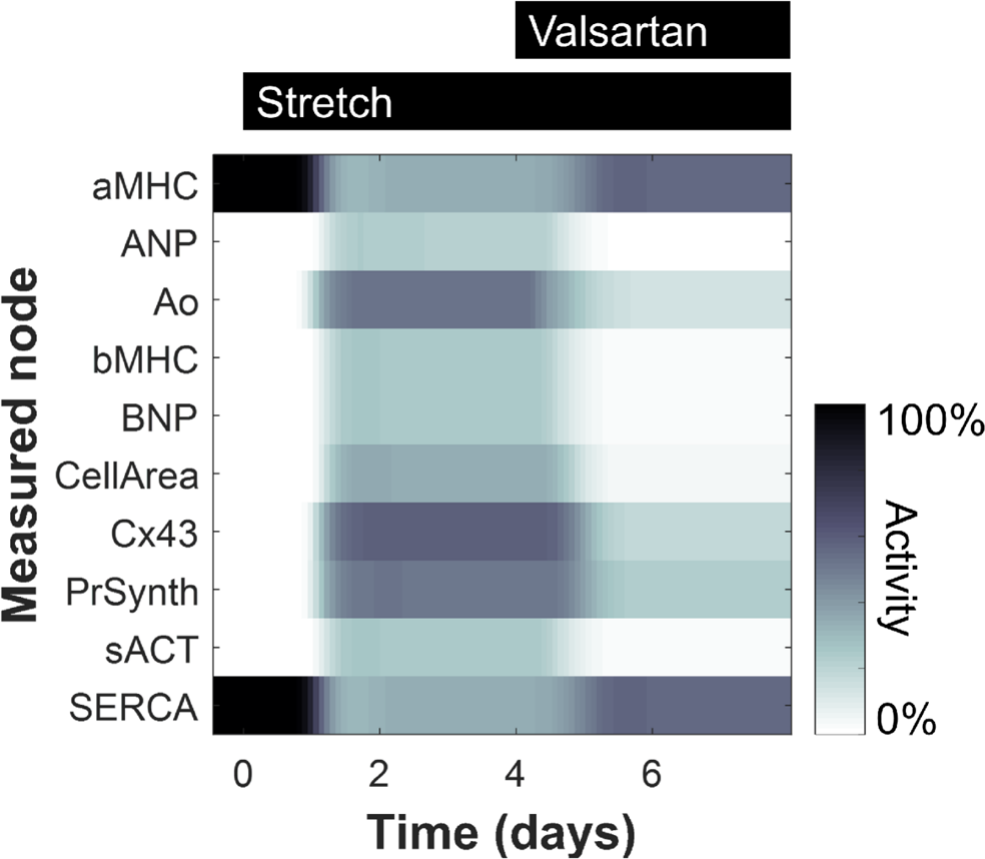
Predicted dynamics of model outputs. Gene expression and phenotype levels are shown for 10 model outputs in response in response to cell stretching (starting at 20 min.) and valsartan (starting at 4 hrs.).

Based on the network structure in S1 Table, the system of LDEs was automatically generated in Netflux and implemented in MATLAB, as detailed in the Methods. A baseline condition of no external stretch is simulated by setting the stretch input at zero, and the response of the network to a high level of stretch can be predicted by increasing the input to 0.7, corresponding to applying approximately a 20% strain to myocytes cultured on a flexible membrane (S1 Fig). In addition, the model can predict the effects on stretch-induced signaling caused by adding an inhibitor against any node in the network. For example, stretch-induced increases in BNP, cell area, and other model outputs are predicted to be partially reduced with the AT1R antagonist valsartan (Fig 2), consistent with previously published results [33–35].

### Model validation and importance of reaction logic

**Fig 3:**
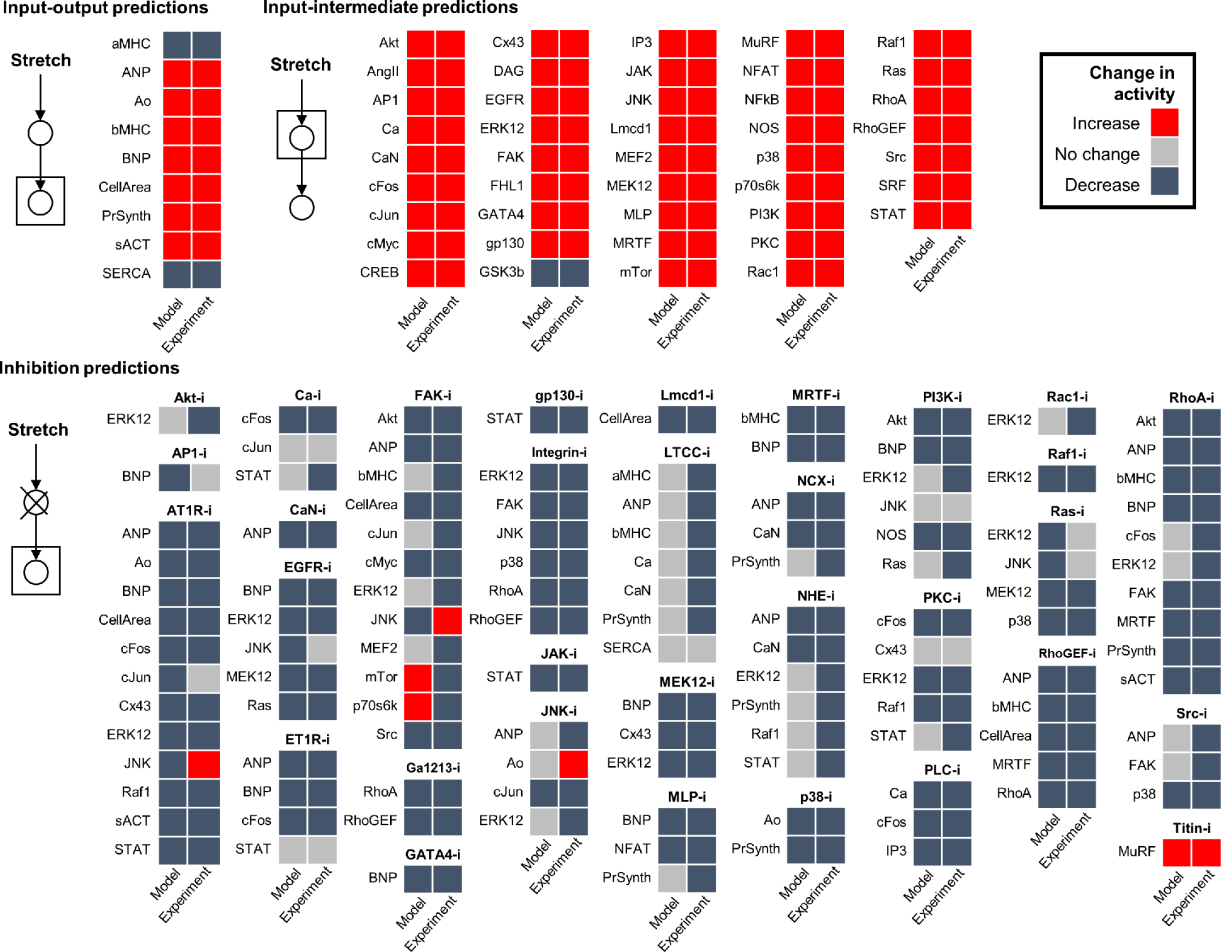
Validation of model predictions against experimental observations not used for model reconstruction. Qualitative activity changes of network nodes were predicted by simulating the response to stretch alone or to stretch together with inhibition of various nodes (first column), and then compared with published experimental observations of *in vitro* rat cardiomyocytes (second column). A validation threshold of 5% relative change was used. Input-output and input-intermediate activity changes are defined relative to no stretch activity, while inhibition activity changes are defined relative to steady-state stretch activation.

To assess the accuracy of model predictions, we simulated activity changes of network nodes in response to stretch alone or to stretch together with inhibition of various nodes, and then compared them with published experimental observations of *in vitro* rat cardiomyocytes. Observations used for validation (S2 Table) included only mechano-signaling experiments performed in rat cardiomyocytes, and were gathered exclusively from literature not used for model construction. Simulated input-output and input-intermediate activity changes were defined relative to no stretch, while inhibition activity changes were defined relative to steady-state stretch. After encoding observations from literature as increase, decrease, or no change, they were compared with model predictions using a 5% threshold for defining change, a more stringent threshold than that of previously published network validations [13,14]. Overall, the model correctly predicts 78% (134/172) of observations from papers not used to construct the model, including 100% (9/9) of input–output predictions, 100% (43/43) of input–intermediate predictions, and 68% (82/120) of inhibition predictions (Fig 3, S2 Table).

To evaluate model robustness to variations in parameters, simulations were tested against parameter sets sampled from uniform random distributions. Consistent with studies of other networks [14,31], validation accuracy is highly robust (>70%) to variation in model parameters over a uniform random distribution of up to ±20% for Y_max_, and up to ±30% or more for all other parameters (S2 Fig). In addition, validation accuracy remains high (>70%) with up to ±30% changes in baseline input levels (S3 Fig).

We also examined whether correct reaction logic is necessary for model accuracy. For example, AND logic was used to model the reaction for BNP, since multiple transcription factors are each necessary (though not individually sufficient) to drive gene expression [36]. In a variation of the model identical to the original but without AND gates (all logic gates set to OR), validation accuracy drops to 51% at the original reaction weight and input levels. Even with reduced reaction weights, the version lacking AND logic cannot validate higher than 70%, and robustness to changes in input level also decreases (S3 Fig), suggesting that logic gating is crucial to proper network function.

### Identification of key network regulators

**Fig 4:**
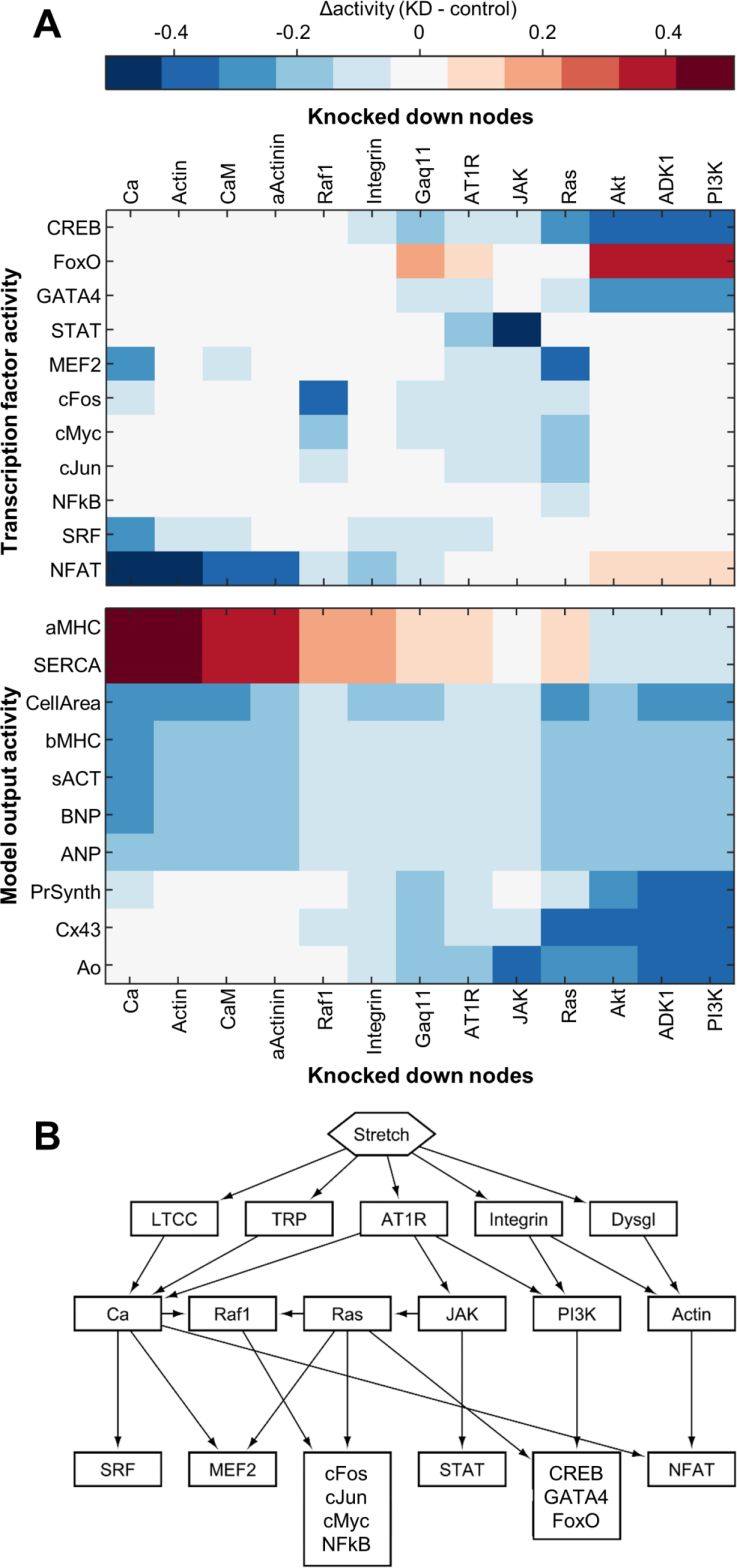
Sensitivity analysis reveals network structure. (A) Network sensitivity to most highly influential hubs. Subset of the sensitivity matrix showing the response of each of the transcription factors and outputs to half-knockdown of each of the 12 nodes causing the highest average response across the transcription factors and outputs, as well as integrin. (B) Simplified network schematic showing control of transcription factors by 6 key hubs.

After validating the model’s predictive capability, we performed a network-wide sensitivity analysis in order to determine quantitative functional relationships across the network. We hypothesized that the structure of the resulting sensitivity matrix would enable identification of key hubs regulating transcriptional activity. Knockdown of individual nodes was simulated by reducing Y_max_ for that node, and the resulting change in activity of every other node was measured, thus predicting the response of the network to inhibition of specific receptors, kinases, or genes. Influential nodes were defined as those whose knockdown causes the greatest activity changes across a given portion of the network. Based on the network-wide sensitivity analysis (S4 Fig), we identified the 15 nodes with the highest influence over transcriptional activity and over the gene expression outputs (Fig 4A). These most influential nodes encompass proteins mediating signals from each of the primary mechano-sensors: Ca^2+^ and calmodulin, downstream of the stretch-sensitive ion channels; Gα_q/11_, which transmits signals from AT1R; and actin and α-actinin, which relay forces from integrins and the dystrophin–dystroglycan complex. Also highly included are previously identified central network hubs for biochemically-stimulated hypertrophy, such as Ras and PI3K. Rather than being controlled by one specific mechano-sensor, most of the hypertrophic outputs display sensitivity to all the stretch-responsive pathways (Fig 4A, lower panel).

In contrast to the outputs, which tend to be broadly sensitive to perturbations in many different parts of the network, most of the transcription factors display sensitivity only to certain mechano-signaling pathways (Fig 4A, upper panel). For example, CREB, FoxO, and GATA4 are primarily regulated by AT1R through the PI3K/Akt pathway, while cFos activity is specific to Raf1 signaling through MEK1/2. To systematically determine the control structure underlying differential control of transcriptional activity, we performed hierarchical clustering on the reduced sensitivity matrix shown in Fig 4A. Using a distance criterion of 0.3 to form groups revealed six clusters, each of which regulates a distinct set of transcription factors. We identified the topologically highest node from each cluster, and then used this to create a simplified network schematic demonstrating how these key hubs—calcium, actin, Ras, Raf1, PI3K, and JAK—link the mechano-sensors to the transcription factors (Fig 4B). Of these six hubs, two are influenced by the mechano-sensitive calcium channels (TRP and LTCC), two are influenced by the cytoskeletal mechano-sensors (integrin and dystroglycan), and five are influenced by AT1R.

### Screen for combination mechano-therapies

**Fig 5:**
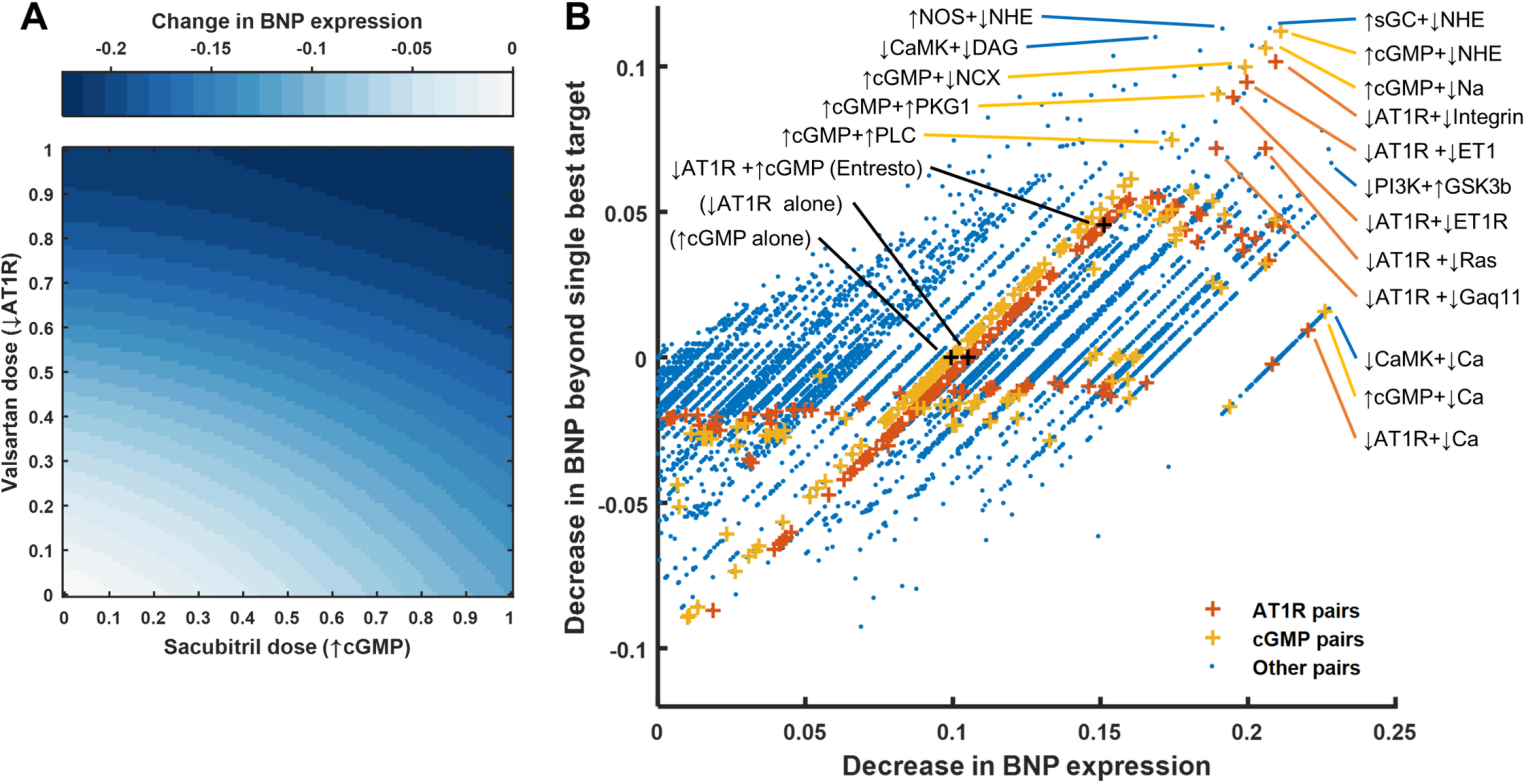
Efficacy of combination mechano-therapies. (A) Response of BNP to increasing doses of valsartan (simulated by progressive inhibition of AT1R) and sacubitril (simulated by progressive activation of cGMP though sGC) in the context of steady-state stretch activation. (B) All pairwise combinations of reducing or increasing Ymax which lowered BNP expression. The x-axis shows the change in BNP relative to steady-state stretch activation, and the y-axis shows the difference between this change and the larger of those caused by targeting either node independently.

While we predicted several individual regulators whose inhibition could reduce stretch-induced gene expression, combination therapies may outperform individual perturbations administered in isolation [37]. For example, the FDA recently approved valsartan/sacubitril (initially known as LCZ696 and branded as Entresto) for treating heart failure [38,39]. Both components of this combination drug affect pathways known to be mechano-sensitive: valsartan inhibits AT1R, and sacubitril increases cGMP by inhibiting neprilysin and thus reducing natriuretic peptide degradation. However, neither the combined effects of these two components on stretch-induced signaling, nor the effect of sacubitril alone, have been assessed to date. To examine valsartan/sacubitril’s influence on cardiac mechano-signaling, we simulated the response to varying levels of valsartan and sacubitril both separately and together. Sacubitril’s anti-hypertrophic effects result from cGMP activating PKG1, which inhibits several different calcium channels and the downstream calcineurin/NFAT pathway (S5 Fig). The model predicts that valsartan/sacubitril will attenuate stretch-induced hypertrophy in myocytes at lower concentrations than either of its individual components (Fig 5A).

Given the predicted benefits of valsartan/sacubitril, as well as the power of systems analysis of drug interactions to uncover network function [40], we were interested in exploring the potential for other drug pairs to reduce mechanically driven hypertrophy. To identify other mutualistic combinations, we ran a sensitivity analysis simulating all pairwise combinations of inhibiting or activating every node in the network, and compared their inhibitory power to that of targeting single nodes (results for BNP shown in Fig 5B). Many of these combinations have additional benefit over single perturbations, including several other combinations with angiotensin receptor blockers. These include inhibiting ET1R, Ras, or integrin signaling simultaneously with AT1R inhibition. The highest-scoring combinations also include several pairings with drugs increasing cGMP, such as those inhibiting NHE or NCX (sodium–calcium exchanger). Other upregulated members of the fetal gene program followed similar patterns to those for BNP, each sharing at least 72% of the top 50 combinations with highest additional benefit.

## Discussion

### Cardiac mechano-signaling model

The high degree of redundancy and crosstalk [7] between stretch-sensitive pathways in the heart renders a systems approach invaluable for identifying mechanisms of signal integration. By developing and validating a comprehensive literature-based reconstruction of the cardiac mechano-signaling network, we demonstrated how network logic and crosstalk between signaling pathways enable cardiomyocytes to integrate distinct mechanical stimuli into a coherent response. Our model, which incorporates five primary mechano-sensors and 94 mechano-responsive nodes connected by 125 reactions, identified calcium, actin, Ras, Raf1, PI3K, and JAK as key regulators of mechanical cues. Although each of these hubs operates through distinct sets of transcription factors, all are crucial for stretch-induced cellular remodeling and activation of the fetal gene program. We also revealed a PKG-dependent mechanism contributing to the mutualistic action of the combination drug valsartan/sacubitril, and predicted further pairs of drug targets with maximum effects on mechano-signaling.

### Model validation

Observations from literature not used in network construction confirmed 78% of model predictions, and the validation rate remained high across wide range of random variation in multiple model parameters. Of the 38 disagreements, the most common (18 instances) were due to the model correctly predicting a change in response to inhibition that was observed in the literature (e.g., a decrease in stretch-induced ANP expression caused by LTCC blockade), but at a magnitude below the 5% threshold. In these cases, more influence could be given to LTCC by modulating the relative weights of downstream reactions within the model to bring the response magnitude above the threshold. Other discrepancies involved inhibitory effects observed in the literature where no connection exists in the model (9 instances), such as lowered stretch-induced Ras phosphorylation in response to PI3K inhibition, or inhibitory effects predicted in the model that were not observed in the literature (7 instances), such as lowered stretch-induced ERK1/2 activity after Ras inhibition. These points of disagreement highlight specific areas where future model revision or further experiments are necessary.

### Key hubs integrating mechano-signals

A longstanding question in cardiac mechanotransduction has been whether the diverse array of stretch-induced signaling pathways function independently or synergistically [41]. Our sensitivity analysis found that while the various pathways maintain mostly independent control over distinct groups of transcription factors, synergy between multiple pathways is necessary to activate all the transcription factors necessary for gene transcription and hypertrophy. Hierarchical clustering based on our sensitivity analysis identified calcium, actin, Ras, Raf1, PI3K, and JAK as the key network hubs integrating signals from the mechano-sensors. Rather than being concentrated in a single pathway, these most influential nodes are distributed across the network and integrate stretch signals from all five primary mechano-sensors. These results help explain why modeling network connectivity and logic correctly is essential for successfully predicting myocyte sensitivity to modulation of a diverse array of stretch-activated pathways.

### Synergistic targets regulate stretch-induced hypertrophy and gene expression

Inhibiting neprilysin counters wide-ranging effects of neurohormonal overactivation such as vasoconstriction and sodium retention, and angiotensin receptor blockers (ARBs) can reduce blood pressure without the angioedemic effects of angiotensin-converting–enzyme (ACE) inhibitors [38]. Here, however, we were particularly interested in how these two interventions could modulate mechano-signaling in cardiomyocytes. Multiple studies have shown that ARBs can attenuate stretch-induced signaling in cardiomyocytes [34,42,43], but a corresponding function for neprilysin inhibition has not been examined either by itself or together with ARBs. We identified a mechano-inhibitory role of the neprilysin inhibitor sacubitril in blocking stretch-sensitive calcium channels with PKG1 by increasing cGMP levels through increased natriuretic peptide receptor stimulation. Our model also predicts that the valsartan and sacubitril reduce hypertrophy more in combination than on their own.

Analysis of all pairs of targets in the network revealed hundreds of potential combinations which inhibit mechano-signaling more significantly in tandem than individually. The high levels of additional inhibition predicted from targeting two nodes simultaneously underscore the importance of a systems pharmacology perspective for crafting new therapies, rather than merely attempting to target the single most important mechano-sensor [44]. Although few of these combinatorial perturbations have previously been tested in the context of cardiac mechano-signaling, the available evidence concurs with our results. For example, the model predicts that inhibiting AT1R and ET1R together should reduce BNP secretion more than inhibiting either individually, and this outcome has been confirmed both in stretched cardiomyocytes [28] and in rats induced with volume overload [43]. Many of the highest changes predicted involve other pairs targeting AT1R or cGMP, suggesting that other drug combinations involving valsartan or sacubitril would be worth pursuing experimentally.

### Limitations and future directions

While the scope of the network reconstruction necessitated the use of default parameters, refinement of parameter weighting as more data becomes available can increase model accuracy. To further enrich the model, future curation could incorporate paracrine signaling from mechanically activated fibroblasts [14], juxtacrine signaling through cadherins [45], more complex autocrine feedback [46], and interaction with related signaling cascades, such as the beta-adrenergic network [30]. Integrating biophysical mechanisms such as force propagation, diffusion, and electrophysiology, which are not directly represented in the current model, could also prove fruitful [15,17,19,47].

Our work also highlights critical gaps in the current understanding of cardiac mechano-signaling. Although the five primary mechano-sensors in the model have each been verified as immediately responsive to mechanical strain, it is unclear whether the activation of several other “stretch receptors” is direct or indirect. For example, there is broad agreement that NHE mediates stretch-dependent signals [26], but it remains controversial whether the role of NHE is dependent on both AT1R and ET1R [42,48], on ET1R alone [49], or on neither [50,51]. Likewise, activation of gp130 and autocrine release of Ang II and ET-1 have all been implicated as contributors to stretch-induced signaling [25,27,28], but the direct cause of each of these effects remains unknown. As others have noted [11], more work is needed to discern which “stretch receptors” are indeed directly responsive to mechanical strain, and which are activated indirectly.

### Conclusions

We developed a large-scale predictive model of cardiac mechano-signaling that identifies the nodes and network structures regulating the response to stretch in cardiomyocytes. Sensitivity analysis of our manually curated network showed that rather than a single stretch sensor governing the response to mechanotransduction, coordination is likely necessary between AT1R, cytoskeletal proteins, and stretch-sensitive ion channels to induce gene expression and hypertrophy. The model also predicts that calcium, actin, Ras, Raf1, PI3K, and JAK are each key hubs with distinct signatures of transcriptional regulation. In addition, we found that network logic is essential for allowing gene expression to be sensitive to a diverse array of mechano-sensors. Our approach integrates results from hundreds of past studies into a coherent model, revealing network interactions unapparent from studying any one pathway in isolation.

## Materials and methods

### Model construction

A predictive computational model of the mechano-signaling network in cardiac myocytes was manually reconstructed from experimental studies described in published literature. To reconstruct the cardiomyocyte mechano-signaling network, experimental observations were synthesized from over 170 peer-reviewed papers. The literature search began by identifying papers that indicated a role for certain proteins in cardiac mechanotransduction, whether in the context of *in vivo* pressure overload or *in vitro* cardiomyocyte stretching. Individual reactions between mechano-responsive proteins were then included if other papers could be found (not necessarily in a mechanotransduction context) confirming a direct molecular interaction between them. During literature review, all papers involving *in vitro* cell-stretching experiments performed in rat cardiomyocytes were set aside for validation. Primary mechano-sensors were included only if evidence from at least three separate studies existed in which either that particular mechano-sensor alone was stretched, or if the mechano-sensor was reconstituted in a cell type previously unresponsive to stretch. Other nodes were only included if identified as mechano-responsive, or if necessarily inferred between other nodes. Outputs were selected for frequency of measurement across the literature and relevance to cardiac function.

Signaling dynamics were predicted with a logic-based differential equation (LDE) approach, in which activation of one node by another is modeled using a normalized Hill function. Logical AND or OR operations were used to represent pathway crosstalk, using the equation *f*(*x*)*f*(*y*) for AND gating and *f*(*x*) + *f*(*y*) − *f*(*x*)*f*(*y*) for OR gating [30]. In general, OR gating is used when each input to a node is sufficient but not necessary for activation, whereas AND gating is used when each input is necessary. Default reaction parameters included Hill coefficient **n** = 1.4 and half-maximal effective concentration **EC**_**50**_ **= 0.5**. Default node parameters included initial activation **Y**_**init**_ = 0, maximal activation **Y**_**max**_ = 1, and time constant **τ** = 1. Logic decisions were primarily made using known biochemical mechanisms, but sometimes inferred from comparing experiments in the literature. The system of LDEs was generated in Netflux (available at https://github.com/saucermanlab/Netflux) and implemented in MATLAB. The input value of 0.7 weight and the weight **w** = 0.9 for other nodes was chosen to maximize the number of nodes activated between 50 and 95%, thus preventing undersaturation or oversaturation in order to obtain the most information from the sensitivity analysis.

### Model validation

Qualitative activity changes of network nodes were predicted by simulating the response to stretch alone or to stretch together with inhibition of various nodes, and then comparing with published experimental observations of *in vitro* rat cardiomyocytes. Observations used for validation were exclusively from literature not used for model construction and only included mechano-signaling experiments performed in rat cardiomyocytes (mostly neonatal ventricular myocytes, with a few studies using adult ventricular or neonatal atrial myocytes). Input-output and input-intermediate activity changes were defined relative to no stretch, while inhibition activity changes were defined relative to steady-state stretch. Observations were encoded as increase, decrease, or no change and were compared with model predictions using a threshold of 5% absolute change, a more robust threshold than that used in previous studies[13,14].

### Parameter robustness

Network robustness to variation in model parameters was tested, using a validation threshold of 5% absolute change. For each parameter shown (**Y**_**max**_, **w**, **n**, and **EC**_**50**_), new values for every instance of that parameter were generated by sampling from a uniform random distribution with indicated half-width about the original parameter value. 100 new parameter sets were created for each distribution range for each parameter, and simulations were run to compare model predictions with literature observations. No changes in validation accuracy resulted from varying **τ** or **Y**_**init**_. Robustness to simultaneous changes in overall reaction weight and weight of initial stretch input were also simulated across the ranges shown.

### Sensitivity analysis

Sensitivity analysis was performed with knockdown simulations run in MATLAB by setting each **Y**_**max**_ to 50% of the default value and measuring the resulting change in activity of every other node compared to steady state activation. Included in the top 12 most influential nodes are the 9 with the highest influence over the transcription factors (Akt, AT1R, Ca^2+^, Gα_q/11_, JAK, PDK1, PI3K, Raf1, and Ras) and the 9 with the highest influence over the outputs (α-actinin, actin, Akt, AP1, Ca^2+^, calmodulin, PDK1, PI3K, and Ras). Hierarchical clustering of this subset of the sensitivity matrix (columns with 12 most influential nodes versus rows with transcription factors and outputs) was performed in MATLAB using Euclidean distance metrics and the unweighted average distance algorithm using a distance criterion of 0.3 to separate clusters. The topologically highest node from each cluster was identified, and grouping of transcription factors was performed by hierarchical clustering of the subset of the sensitivity matrix comprising columns with the 12 most influential nodes and rows with the transcription factors, using the same settings as before.

Double sensitivity analysis was run by measuring the network response to all pairwise combinations of decreasing or increasing **Y**_**max**_ by 50% of its original value. Additional effects of pairs of nodes were measured by subtracting the higher sensitivity value due to decrease (or increase) of either node individually from the sensitivity due to decrease (or increase) of both nodes simultaneously.

## Supporting information

S1 Table Mechano-signaling network model. This database includes information about each species and each reaction in the cardiac mechano-signaling network, as well as references used in model construction.
(XLSX)

S2 Table Validation relationships. This database includes a list of activity changes predicted by the model, as well as references used for experimental validation.
(XLSX)

S3 Table Experimental parameters. This database summarizes parameters for the cell stretching experiments from the literature used for model construction or validation.
(XLSX)

S1 Fig Simulated activation of the cardiac mechano-signaling network. The steady-state response to a stretch input of 0.7 is displayed.
(TIF)

S2 Fig Network robustness to variation in model parameters. 100 new parameter sets were created for each distribution range for each parameter, and simulations were run to compare model predictions with literature observations, using a validation threshold of 5% absolute change. For each parameter tested (Y_max_, w, n, and EC50), new values for every instance of that parameter were generated by sampling from a uniform random distribution with indicated half-width about the original parameter value. (No changes in validation accuracy occurred in response to varying tau or y0.)
(TIF)

S3 Fig Influence of model logic on prediction accuracy. (a) Prediction accuracy of the original model. (b) Prediction accuracy of a model version with all activating AND reactions converted to OR reactions. For each version, network validation was tested across a range of initial stretch inputs (from 0.10 to 1.0) and default reaction weights (from 0.7 to 1.0), using a validation threshold of 5% absolute change.
(TIF)

S4 Fig Network-wide sensitivity matrix. The matrix displays the sensitivity of each node to all other nodes in the context of steady-state stretch activation. Each column of the matrix represents a simulation in which one node was knocked down 50% and the change in activation of every other node in the network was measured.
(TIF)

S5 Fig Network response to valsartan and sacubitril individually and combined. Response of network to valsartan (simulated by progressive inhibition of AT1R), sacubitril (simulated by progressive activation of cGMP through sGC), and the combination of valsartan and sacubitril, all in the context of steady-state stretch activation.
(TIF)
