# Supplementary figures and images for: Predictive model identifies key network regulators of cardiomyocyte mechano-signaling
Source: PLoS Comput Biol. 2017 Nov 13;13(11):e1005854. doi: 10.1371/journal.pcbi.1005854 (PMC5703578; doi:10.1371/journal.pcbi.1005854)

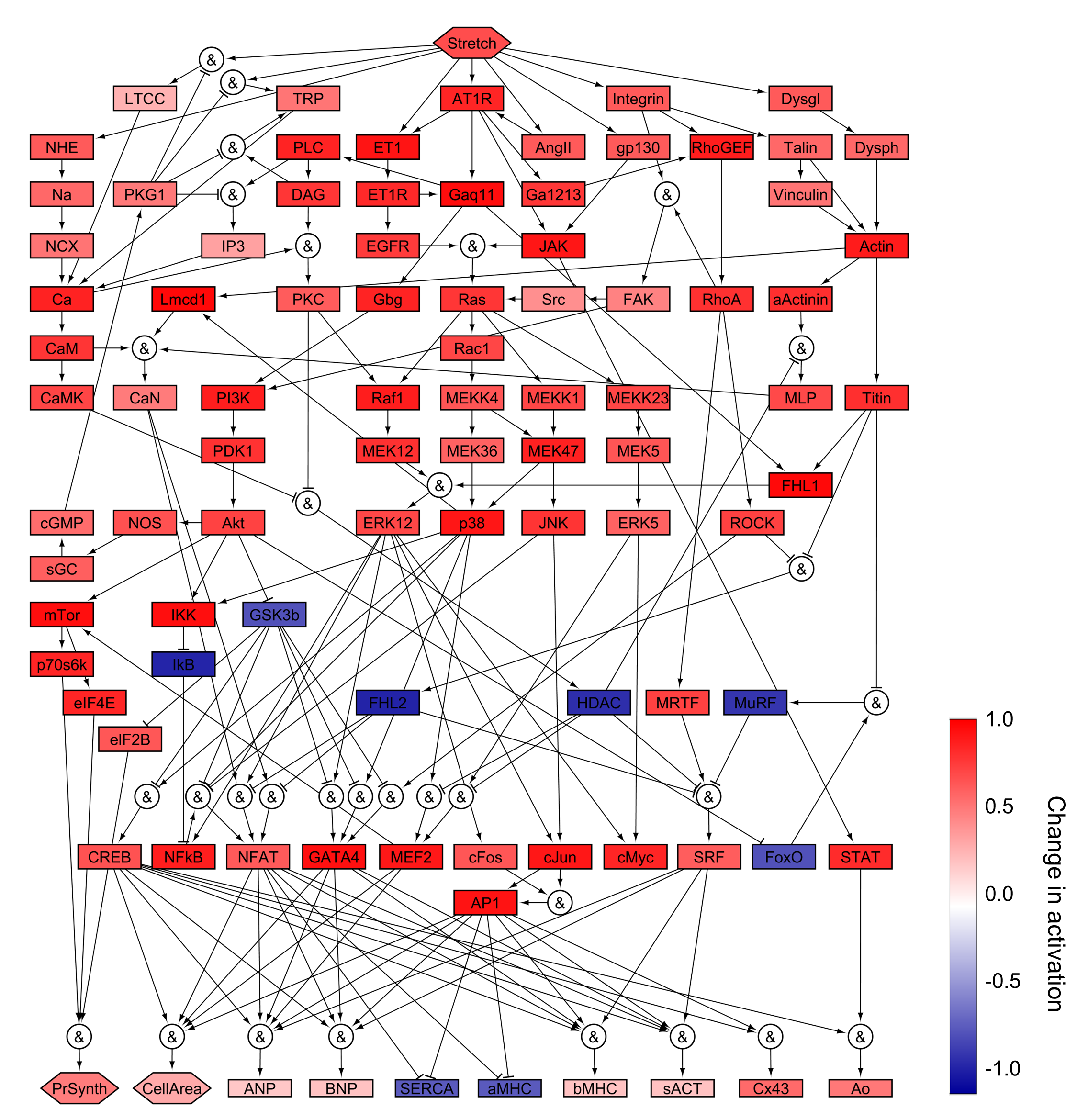

Supplement: S1 Fig — The steady-state response to a stretch input of 0.7 is displayed. (TIF) [file pcbi.1005854.s004.tif]

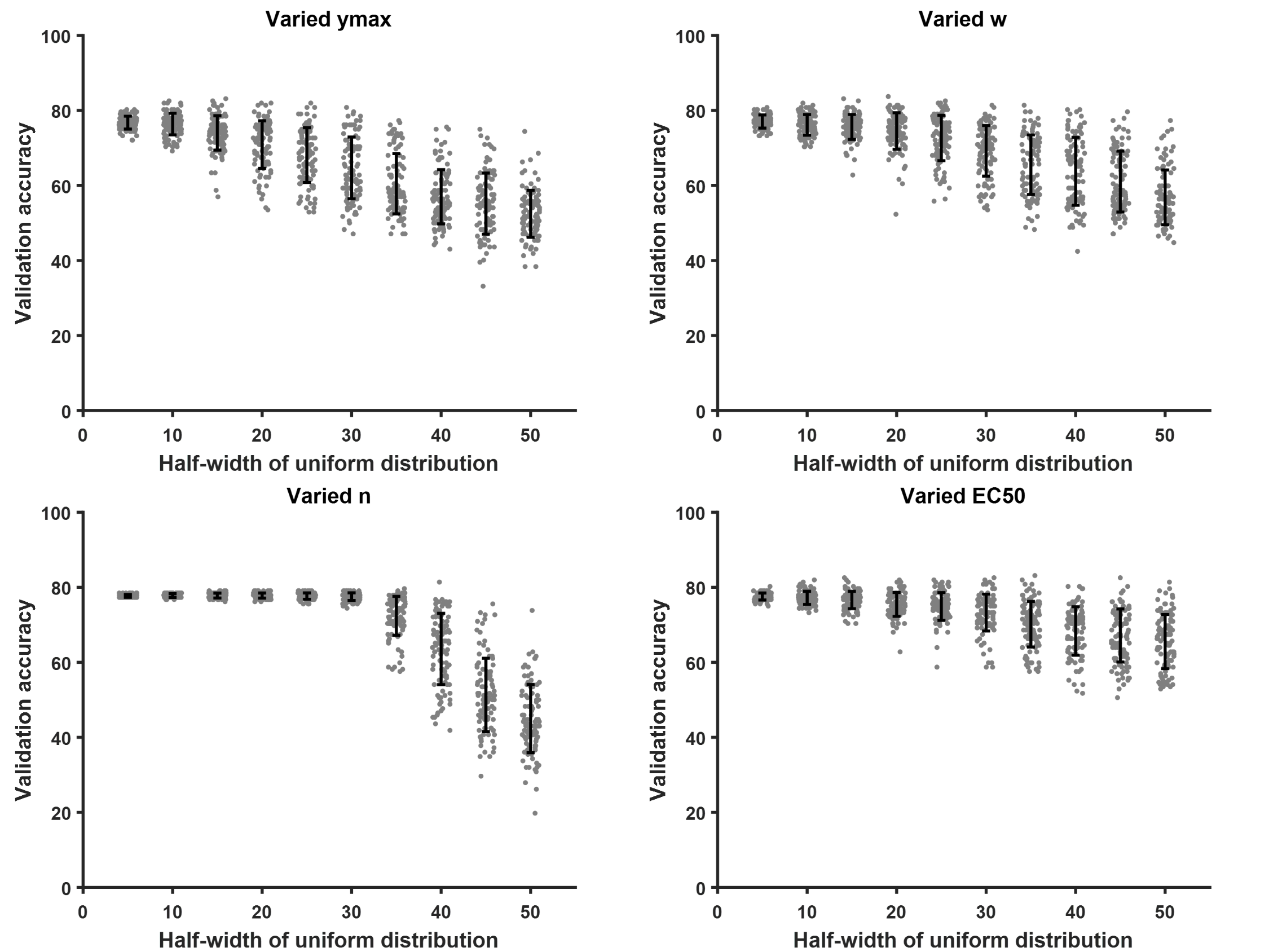

Supplement: S2 Fig — 100 new parameter sets were created for each distribution range for each parameter, and simulations were run to compare model predictions with literature observations, using a validation threshold of 5% absolute change. For each parameter tested (Ymax, w, n, and EC50), new values for every instance of that parameter were generated by sampling from a uniform random distribution with indicated half-width about the original parameter value. (No changes in validation accuracy occurred in response to varying tau or y0.) (TIF) [file pcbi.1005854.s005.tif]

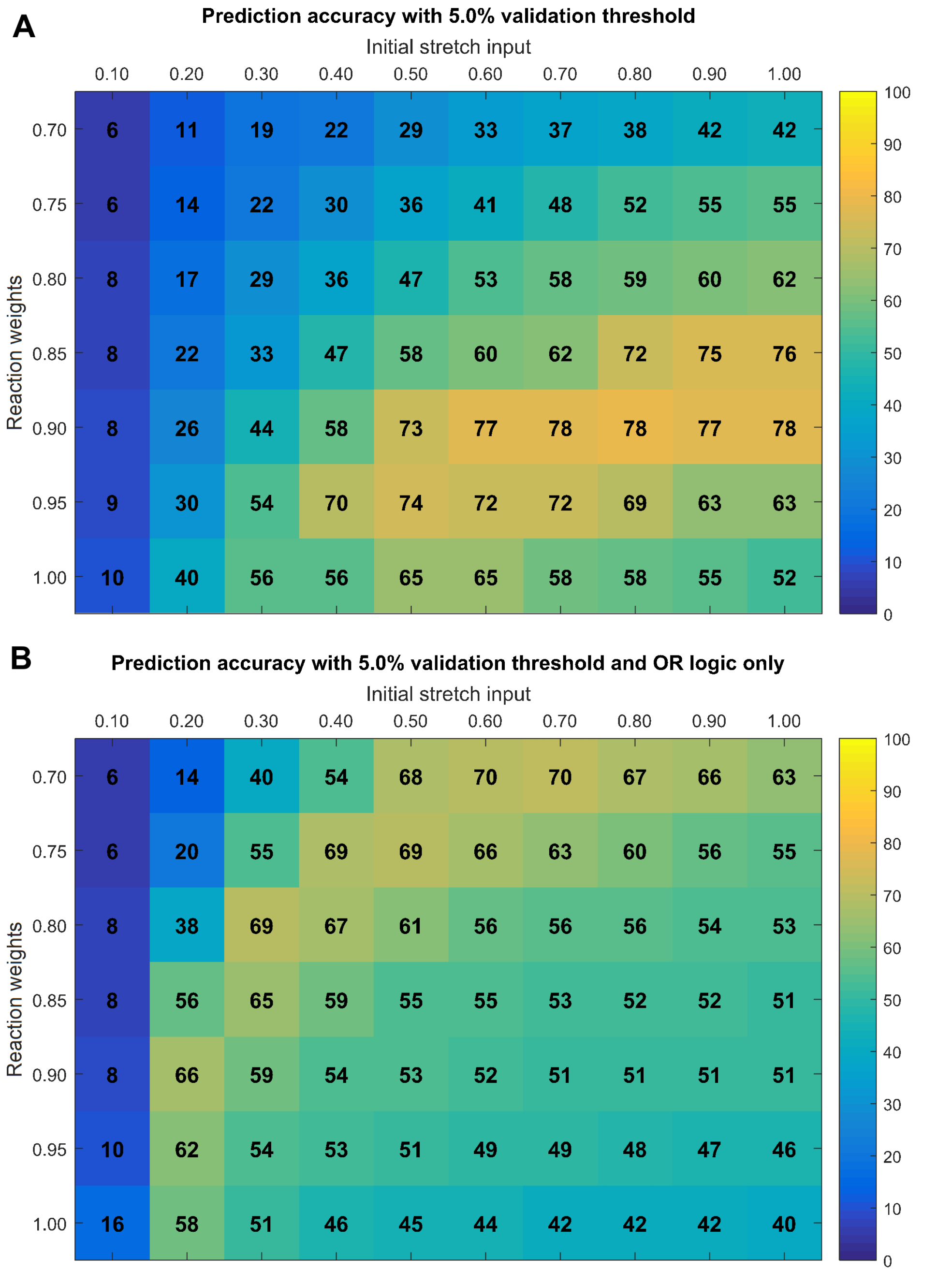

Supplement: S3 Fig — (a) Prediction accuracy of the original model. (b) Prediction accuracy of a model version with all activating AND reactions converted to OR reactions. For each version, network validation was tested across a range of initial stretch inputs (from 0.10 to 1.0) and default reaction weights (from 0.7 to 1.0), using a validation threshold of 5% absolute change. (TIF) [file pcbi.1005854.s006.tif]

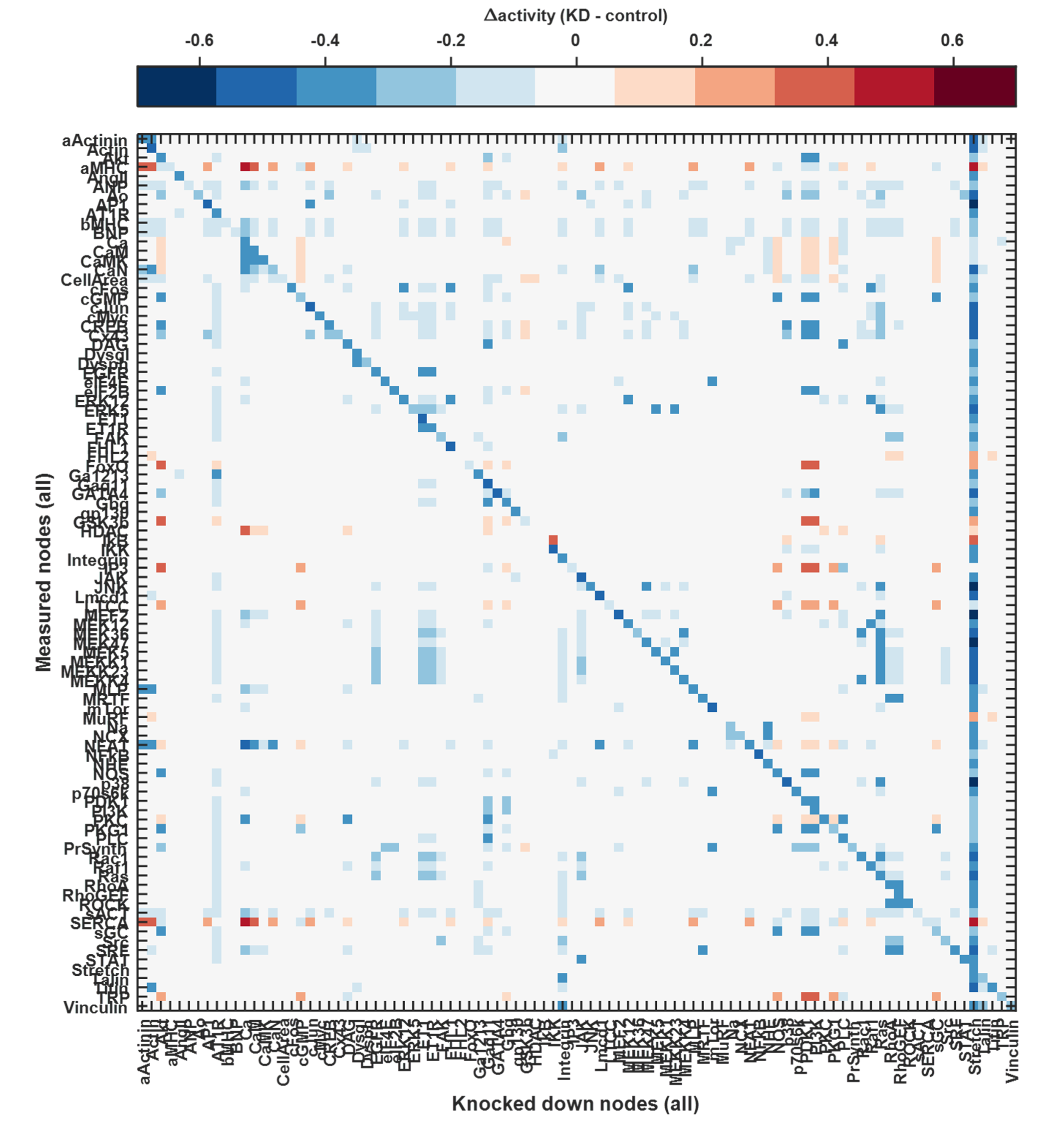

Supplement: S4 Fig — The matrix displays the sensitivity of each node to all other nodes in the context of steady-state stretch activation. Each column of the matrix represents a simulation in which one node was knocked down 50% and the change in activation of every other node in the network was measured. (TIF) [file pcbi.1005854.s007.tif]

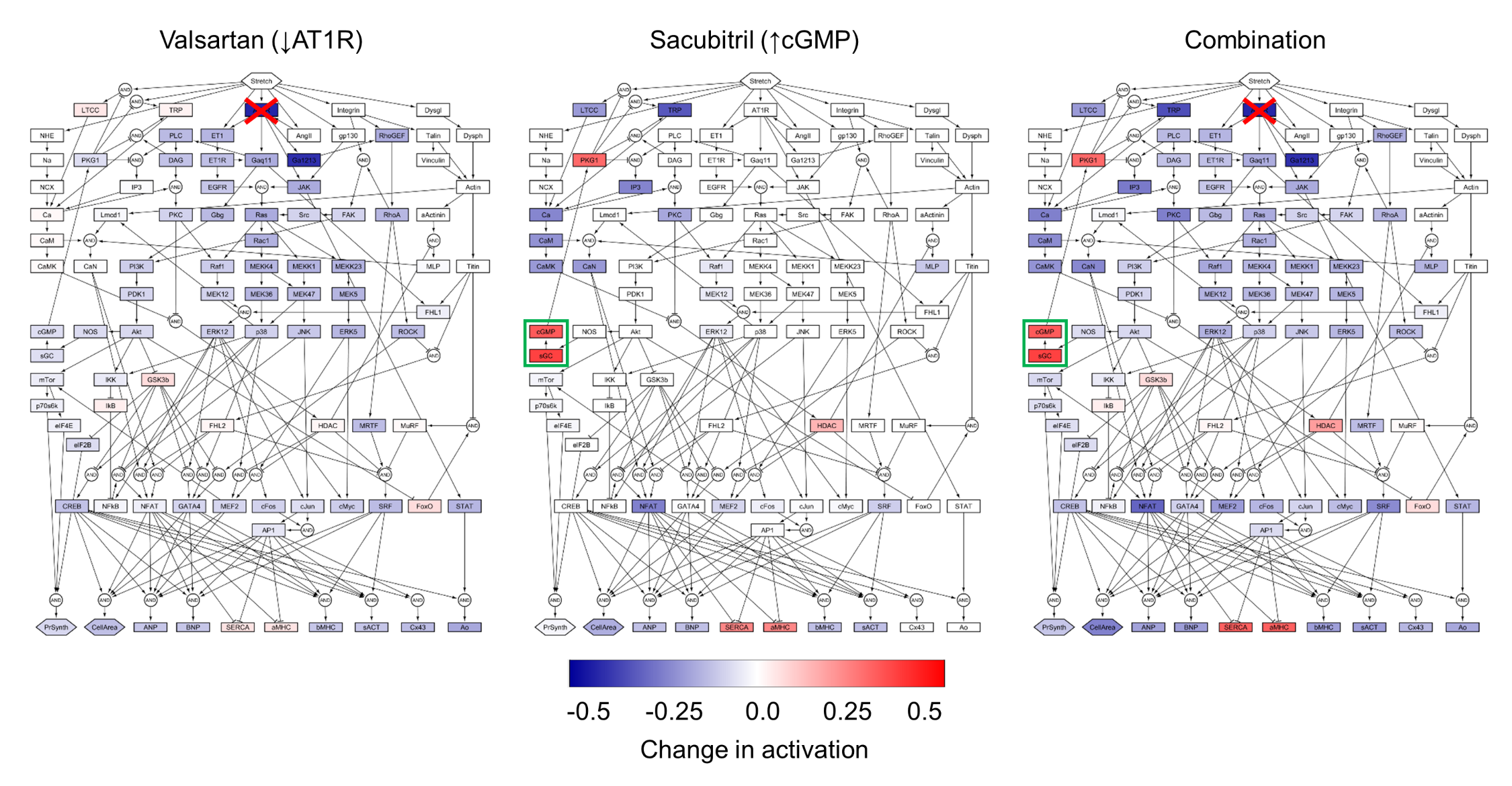

Supplement: S5 Fig — Response of network to valsartan (simulated by progressive inhibition of AT1R), sacubitril (simulated by progressive activation of cGMP through sGC), and the combination of valsartan and sacubitril, all in the context of steady-state stretch activation. (TIF) [file pcbi.1005854.s008.tif]
